# Nevertheless, the importance of coagulation abnormalities should be emphasized in international sepsis guidelines

**DOI:** 10.1186/s40560-022-00596-6

**Published:** 2022-01-21

**Authors:** Toshiaki Iba, Osamu Nishida, Jerrold H. Levy, Marcel Levi

**Affiliations:** 1 Department of Emergency and Disaster Medicine, Juntendo University Graduate School of Medicine, 2-1-1 Hongo Bunkyo-ku, Tokyo, 113-8421 Japan; 2 Department of Anesthesiology and Critical Care Medicine, Fujita Health University School of Medicine, Kutsukake-Machi, Toyoake, 471-11 Japan; 3 Department of Anesthesiology, Critical Care, and Surgery, Duke University School of Medicine, Durham, NC USA; 4 Department of Medicine, University College London Hospitals NHS Foundation Trust, and Cardio-Metabolic Programme-NIHR UCLH/UCL BRC London, Tottenham Court Road, London, UK; 5 Department of Vascular Medicine, Amsterdam Cardiovascular Science, Amsterdam University Medical Centers, Amsterdam, the Netherlands

**Keywords:** Sepsis, Coagulopathy, Disseminated intravascular coagulation, Endothelial cell, Anticoagulation

## Abstract

It is generally accepted that a coagulation/fibrinolysis disorder is involved in the pathogenesis of sepsis, and the association of disseminated intravascular coagulation (DIC) and poor outcomes have been reported. Based on these findings, recently released “Japanese Surviving Sepsis Campaign guidelines 2020” recommend the diagnosis of DIC and the application of anticoagulants for sepsis-associated DIC. Meanwhile, the updated “International Guidelines for the Management of Sepsis and Septic Shock 2021” did not mention coagulation abnormalities or DIC. Because management strategies continue to evolve to provide improved outcomes in sepsis, the role of adjunctive anticoagulant treatment should be included in subsequent international guidelines.

## Dear Editor,

We realized that there are some discrepancies between the recently updated “Japanese Surviving Sepsis Campaign guidelines 2020” and “Surviving Sepsis Campaign International Guidelines for the Management of Sepsis and Septic Shock 2021” [1, 2]. In the Japanese guidelines, the early detection of disseminated intravascular coagulation (DIC) is emphasized and the use of certain anticoagulants for sepsis-associated DIC was weakly recommended. Meanwhile, the chapter on “anticoagulation” is eliminated from the recent version of International guidelines.

It is widely accepted that an extensive connection and cross-talk between inflammation and coagulation occurs in sepsis. Indeed, the mortality of sepsis worsens considerably when a patient develops coagulation abnormalities, in its most extreme form DIC. Although mechanistically different, COVID-19 taught us that coagulopathy and subsequent pulmonary and multiorgan microthrombosis are important in the pathogenesis of organ failure and death in thromboinflammatory diseases [3] as well as the occurrence of venous thromboembolism. As a reminder, coagulopathy that occurs in acute infectious processes represents acute inflammation, dysregulated immune reactions, platelet activation, and endothelial dysfunction [4]. For these reasons, anticoagulation is important for preventing thromboembolism but also microthrombosis in COVD-19 but also in sepsis-induced coagulopathy. Apart from that, antithrombotic prophylaxis is crucial to prevent thromboembolic complications in critically ill patients.

The early detection of coagulopathy is critical and Umemura et al. [5] reported the screening of DIC improved the outcome in patients with sepsis. Although no consistently reliable method is available to evaluate the coagulopathy in sepsis, the International Society on Thrombosis and Haemostasis introduced a scoring system for sepsis-induced coagulopathy (SIC), and emphasized the importance of early detection of a coagulation derangement [6]. SIC consists of a simple algorithm (platelet count, prothrombin time [international normalization ratio], and Sequential Organ Failure Assessment [SOFA] score) and has been validated to detect the coagulopathy in sepsis.

Although there is no proven effective treatment for coagulopathy and DIC in sepsis, Japanese Surviving Sepsis Campaign guidelines 2020 weekly recommend the use of antithrombin and recombinant thrombomodulin for DIC. Since some of the authors have a conflict of interest with the related pharmaceuticals, it may not be appropriate to discuss the effects of anticoagulation in this letter. However, it is necessary to explain that although a recent randomized controlled trial examining the effect of recombinant thrombomodulin in severe sepsis (SCARLET trial) failed to show a beneficial effect on survival in an intention-to-treat population, it showed a survival benefit in patients with more severe coagulopathy [7]. Levi et al. [8] also demonstrated that patients treated with recombinant human soluble thrombomodulin having higher baseline prothrombin fragment _1.2_ or thrombin–antithrombin complex had lower mortality compared with patients receiving placebo in post hoc analysis. Furthermore, other anticoagulant interventions such as supplementation of antithrombin and restoration of glycocalyx have shown promising results in patients with severe sepsis and coagulopathy [9, 10].

We hope that in the subsequent version of the international guidelines, consideration of the role of coagulation and appropriate anticoagulant adjunctive treatment in severe sepsis will be included to underline the importance of detecting and managing coagulopathy in these critically ill patients.

## Data Availability

Not applicable.

## Abbreviations

DIC: Disseminated intravascular coagulation
SIC: Sepsis-induced coagulopathy
SOFA: Sequential Organ Failure Assessment
